# Prevalence and potential risk factors of flight-related neck, shoulder and low back pain among helicopter pilots and crewmembers: a questionnaire-based study

**DOI:** 10.1186/s12891-019-2421-7

**Published:** 2019-01-29

**Authors:** Markus Posch, Alois Schranz, Manfred Lener, Werner Senn, Björn O. Äng, Martin Burtscher, Gerhard Ruedl

**Affiliations:** 1 Department of Sport Science of the University of Innsbruck, A-6020 Innsbruck, Austria; 2 Medalp Sportclinic, A-6460 Imst, Austria; 3 Federal Ministry of the Interior, Austrian Airborne Police, A-1120 Wien, Austria; 4 Division of Physiotherapy, Department of Neurobiology, Care Sciences and Society, Karolinska Institutet, Huddinge, Sweden; 5 School of Education, Health and Social Studies, Dalarna University, Falun, Sweden

**Keywords:** Neck pain, NVG, Risk factor, Helicopter pilots, Crewmembers, Airborne police, Military, Rescue organizations

## Abstract

**Background:**

Flight-related neck, shoulder and low back pain are the most common musculoskeletal disorders among helicopter pilots and their crewmembers, thus becoming a growing concern. Information on the combined prevalence of these types of pain and related risks are scarce. The aim of this study was therefore to estimate pain prevalence and to evaluate potential risk factors for neck pain among helicopter pilots and crewmembers within the armed forces, the airborne police and airborne rescue organizations in Austria.

**Methods:**

Among a cohort of 104 helicopter pilots and 117 crewmembers (69.8% compliance), demographics, flying experience, use of Night Vision Goggles (NVG), helicopter type flown, prevalence and intensity of musculoskeletal symptoms (pain was defined as any reported pain experience, ache or discomfort) were collected by an online-based questionnaire.

**Results:**

For helicopter pilots the 12-month prevalence of neck pain was 67.3%, followed by low back (48.1%) and shoulder pain (43.3%). Among crewmembers, the 12-month pain prevalence were 45.3, 36.8 and 30.8% among the neck, lower back and shoulder, respectively. During this period, 41.8% of these helicopter pilots had experienced 8–30 pain days in the areas of neck (45.7%), shoulder (37.8%) and lower back (42.0%) whereas 47.8% of crewmembers self-reported 1–7 days of neck (54.7%), low back (44.2%) and shoulder (44.4%) pain in the previous year. The 3-month prevalence of neck pain was 64.4% followed by low back (42.3%) and shoulder pain (38.5%) for helicopter pilots. Among crewmembers, 41.9% suffered from neck, 29.9% from low back and 29.1% from shoulder pain the previous 3 months. Multivariate regression analysis revealed NVG use (OR 1.9, 95% CI, 1.06–3.50, *p* = 0.032), shoulder pain (OR 4.9, 95% CI, 2.48–9.55, *p* < 0.001) and low back pain (OR 2.3, 95% CI, 1.21–4.31, *p* = 0.011) to be significantly associated with neck pain.

**Conclusions:**

The 12- and 3-month prevalence of neck, shoulder and low back is considerably high among both, helicopter pilots and crewmembers confirming the existence of this growing concern. The use of NVG devices, shoulder and low back pain in the previous 12 months represent independent risk factors for neck pain. These findings highlight the need for longitudinal studies.

## Background

As alpine outdoor activities gain increasing popularity [1], emergency medical helicopter services (HEMS) are regularly involved in rescue missions [2]. In Austria, mountain hiking is the most popular mountain sport activity during the summer season and alpine skiing during the winter season, attracting several million hikers and skiers each year [3, 4]. More than 6700 rescue missions are flown by Austrian helicopter pilots and crewmembers of the airborne police and airborne rescue organizations per year (personal communications), constituting high stress on the musculoskeletal system [5].

Chronic exposure to high forces countered by submaximal muscular contractions may lead to musculoskeletal disorders, especially flight-related neck, shoulder and low back pain [5]. While low back pain is a well-documented topic in helicopter pilots with the highest rates of 61–80% [6, 7] among occupations [8], flight-related neck and shoulder pain has not been noticed in scientific literature before the 1990s [9]. At date, neck pain is a growing concern and one of the most common musculoskeletal problems among helicopter pilots and crewmembers [5–7, 10]. Generally, the prevalence of neck pain among fast jet pilots and helicopter aircrew is higher when compared with the general population [11]. Approximately one-third of the general population on average suffer from neck pain and discomfort in a year [11]. Pursuant to results of various studies the point prevalence among military helicopter pilots and crewmembers is as high as 29% [6], comparable to the 12-month prevalence in dutch military helicopter pilots [12]. Äng and Harms-Ringdahl [13] proved the 3-month prevalence to be 57% in Sweden, while in the United Kingdom, the prevalence ranges from 38 to 81% [14]. A recent report by Walters et al. [15] reveals 58% of helicopter aircrew within the United States Army report flight-related neck pain. In Canada, the lifetime prevalence is estimated between 75 and 84% [16, 17], indicating an individual health concern at leisure.

The flight helmet represents an essential component of the aircrew protective equipment [5], by protecting the head from impacts during flying [18, 19]. Headborne equipment becomes more popular as helmets are frequently used as a mounting platform for several combat essential devices like night vision goggles (NVG) [19, 20]. In a study by Harrison et al. [5], NVG is described as a tool to allow pilots and crewmembers to enhance their visual capacity while operating under low light conditions to prevent serious accidents. However, all mounted devices result in higher head-worn mass and simultaneously alter the center of gravity of the helmet [13, 21, 22]. Experimental studies in human centrifuges indicate that increased loading of the cervical spine increases neck muscle strain in stabilizing muscles, indicating that the inertia of head-worn NVG elevates the risk of flight-related neck pain [23], and such cervical loading has been reported as a risk factor for neck pain and discomfort in helicopter pilots and crewmembers as well [5]. Posture, low +G_z_ forces and vibrations while using NVG and an extended period of submaximal loading are perceived causes for flight-related neck pain [24].

Moreover, neck pain potentially influences the level of concentration [6, 13], motor control [25–27], postural stability [28] and finally operational safety [16] of helicopter pilots and crewmembers. As Äng and Harms-Ringdahl [13] found high pain prevalences in other close body regions like the shoulder and lower back too, it is important to further evaluate pain prevalences for the whole body. Despite the great importance of these types of musculoskeletal disorders, literature among this topic is sparse, especially among helicopter aircrewmembers of the airborne police and airborne rescue organizations. The majority of the available literature related to flight and NVG-induced neck pain has focused mostly on military fast-jet aircrew [29–31] and military helicopter pilots [5, 13, 32]. To the best of our knowledge, no study has yet evaluated combined 12- and 3-month pain prevalence for the neck, back and shoulder for Austrian helicopter pilots and crewmembers among several occupational groups.

Therefore, the aim of this study was to estimate general musculoskeletal pain prevalences, particularly focusing on neck, shoulder and low back pain, and to evaluate potential neck pain risk factors among helicopter pilots and crewmembers within the Austrian armed forces, the Austrian airborne police and Austrian airborne rescue organizations.

## Methods

### Study design

This questionnaire-based cross sectional study was conducted within Austrian helicopter pilots and crewmembers from March to May 2018.

### Study participants

Potential study participants were informed about the project and recruited at briefings, by emails and by telephone. Reminders were sent fortnightly over the whole study period (3 months). In total, 221 helicopter pilots and crewmembers from different professional sectors (Austrian armed forces, Austrian airborne police and Austrian airborne rescue organizations) agreed to participate in this study (78.7% of airborne police, 74.5% of armed forces and 56.3% of airborne rescue organizations) with an overall response rate of 69.8%.

Inclusion criteria was a profession as a helicopter pilot or crewmember. Pilots and crewmember were excluded if they had not been on flying duty during the previous 3 months (2.5%).

### Questionnaire

An online-based questionnaire was applied to all participants. The online questionnaire comprised two sections. The first section collected data regarding demographics, flying experience (total flying hours, flying hours in the previous year, average flying hours per month the previous three months), use of NVG (years, hours) and type of helicopter flown according to Äng and Harms-Ringdahl [13]. In the second section, a modified version of the validated Nordic Musculoskeletal questionnaire [33] was used to assess prevalence and intensity of musculoskeletal symptoms (pain) in the following body regions: head, neck, shoulder, upper back, elbow, lower back, forearm, hip, knee and lower leg. In accordance to Murray et al. [32], Äng and Harms-Ringdahl [13] and van den Oord et al. [12] we defined pain as any reported pain experience, ache or discomfort.

According to the Nordic Musculoskeletal questionnaire [33] study participants were asked about pain frequency during the previous 12 months (0 days, 1–7 days, 8–30 days, > 30 days) and intensity of pain previous 3 months (0 = no pain, 10 = worst possible pain imaginable on a 11 point numeric box scale) [34].

As a novelty in our study we additionally asked participants who stated pain in the previous 12 months for pain prevalence among the whole body in the previous 3 months (never, a few times over the previous 3 months, a few times per month, a few times per week the previous 3 months) to get detailed information about musculoskeletal disorders. Furthermore, the short time frame of 3 months was chosen to reduce potential recall bias, since recent and more serious pain episodes may be remembered better than earlier ones [13]. Participants who reported any pain frequency were further asked on pain occurrence (in resting phase, during flying) and on their possible inability to perform activities of daily living or working tasks (interference with flying duty: yes or no; interference with leisure activity: yes or no). For the statistical analysis helicopter pilots and crewmembers were further divided into cases (reporting any neck pain in the previous year) and controls (reporting no neck pain in the previous year).

The online questionnaire was validated preceding the present survey: questions of the online survey were initially generated and identified by a consensus panel (*n* = 3) for collecting sufficient data to measure the content domain [35]. The consensus panel consisted of three of the listed authors (MP; BA; WS). MP is a postdoc researcher in the field of injury prevention, epidemiology and exercise therapy. BA is a biomechanics expert and registered physical therapist. WS is the head of the Austrian airborne police of the federal ministry of the interior and an experienced helicopter pilot.

Nine experienced helicopter pilots (total flying time > 4500 h, mean age 49.3, SD 8.7 years) from different profession sectors (Austrian airborne police *n* = 3, and airborne rescue organizations *n* = 6) were invited to participate as independent expert raters of the online questionnaire. In order to quantify content validity for multi item scales of this questionnaire, the item content validity index (I-CVI) as well as the overall scale content validity index (S-CVI) were computed [35, 36]. Pursuant to Polit et al. and Davis, I-CVI was calculated by asking experts (*n* = 9) to rate the relevance of each item of the questionnaire on a 4-point scale (1 = not relevant, 2 = somewhat relevant, 3 = quite relevant, 4 = highly relevant) followed by iterative loops of consensus panel revisions [35, 37]. S-CVI was evaluated by computing the I-CVI across all items [35, 36]. All items had an I-CVI of 0.83 or higher and S-CVI was 0.90, representing evidence of good content validity [35]. Based on the expert ratings no items had to be discarded or improved and no further revisions were required.

In total, seven flight-related risk variables and seven individual indicators were considered for the use in the risk factor analysis. Flight related risk factors comprised total flying time (h), flying hours in the previous year (h), average flying hours per month in the previous 3 months (h), use of NVG (yes or no), NVG flying hours (h), NVG flying years (years), type of helicopter flown (3 helicopter aircraft categories were used: 1) Augusta Bell 58/206/212/407; 2) Eurocopter 135; 3) Airbus 350/355). Individual indicators consisted of profession (helicopter pilot or crewmember), age (years), weight (kg), height (m), body mass index (BMI, kg/m^2^), shoulder and low back pain (yes or no) in the previous 12 months.

### Statistical analysis

Demographic data of study participants are presented as means and standard deviations as well as absolute and relative frequencies. Factors with more than two categories (helicopter type flown) were binary coded for every single category to achieve univariate odds ratio (OR).

Pursuant to tests of normal distribution (Kolmogorov Smirnov), differences in age were evaluated by independent t-tests, whereas differences in weight, height, BMI, total flying hours, flying hours in the previous year and average flying hours per month in the previous 3 months between helicopter pilots and crewmembers suffering from flight related neck pain (cases) and those not reporting neck pain (controls) were computed by Mann-Whitney-U-Tests.

In addition, according to the univariate results, a binary logistic regression analysis entering all variables with *p* < 0.25 was used to calculate multivariate OR and 95% confidence interval (CI) [13].

SPSS 23.0 (IBM Corporation, Armonk, NY, USA) was used for the statistical analysis. All *p*-values were two-tailed and statistical differences were considered significant at *p* < 0.05.

## Results

A total of 104 helicopter pilots and 117 crewmembers with a mean age of 44.7 (SD, 8.4) years, mean height of 1.80 (SD, 0.1) m, mean body weight of 80.9 (SD, 9.9) kg and a mean BMI of 24.8 (SD, 2.4) kg/m^2^ volunteered to participate in this questionnaire based study. Most study participants worked in the air emergency sector (64.0%), followed by members of the airborne police (27.9%) and the military (8.1%). The most frequent used type of helicopter was the EC 135 (78.4%) followed by AS 305/355 (7.2%) and Augusta Bell 58/206/212/407 (7.2%).

**Fig. 1:**
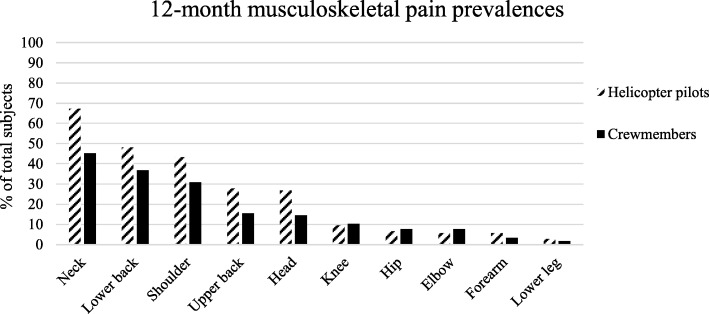
Relative frequencies of 12-month pain prevalence in different body regions

The 12-month prevalence of neck pain was 67.3% (95% CI, 57.3–76.0) for helicopter pilots (Fig. 1), followed by low back (48.1, 95% CI, 38.3–58.1) and shoulder pain (43.3, 95% CI, 33.7–53.3). Of these 31.4% had experienced 1–7 days with neck pain, 45.7% had experienced 8–30 pain days and 22.9% had experienced > 30 days with neck pain in the previous 12 months. Furthermore, the majority of helicopter pilots had experienced 8–30 pain days among the shoulder (37.8%) and lower back (42.0%).

As presented in Fig. 1, within crewmembers the 12-month prevalence was 45.3% (95% CI, 36.2–54.8), 36.8% (95% CI, 28.2–46.2) and 30.8% (95% CI, 22.8–40.1) among the neck, lower back and shoulder. Of these crewmembers, 54.7% had experienced 1–7 days of neck pain, 28.3% had experienced 8–30 days of neck pain and 17.0% had experienced > 30 days of neck pain. In contrast to helicopter pilots, most crewmembers self-reported 1–7 days of shoulder (44.4%) and low back (44.2%) pain in the previous year.

**Fig. 2:**
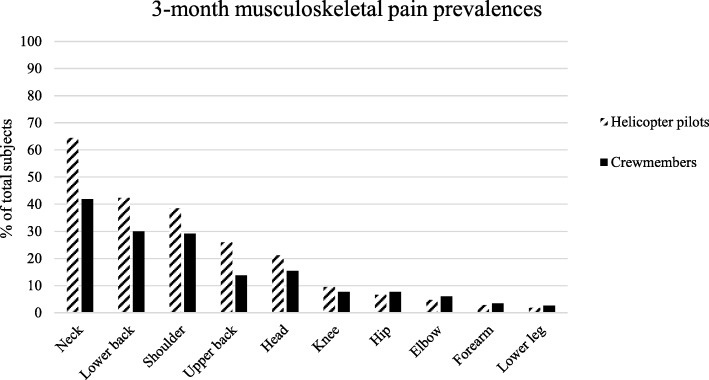
Relative frequencies of 3-month pain prevalence in different body regions

Moreover, 12- and 3-month pain prevalence of helicopter pilots and crewmembers among other anatomical locations are presented in Figs. 1 and 2. For the total group, regardless if considering the 12-month or 3-month prevalence, low back pain presents the second most frequent painful body region followed by the shoulder.

For helicopter pilots the 3-month prevalence of neck pain was 64.4% (95% CI, 54.4–73.4), followed by low back (42.3, 95% CI, 32.8–52.4), and shoulder pain (38.5, 95% CI, 29.2–48.6). Of these helicopter pilots, a minor part suffered from frequent pain (few times per week in the previous three months) among the neck (13.4%), shoulder (12.5%) and lower back (13.6%).

Most crewmembers suffered from neck pain (41.9, 95% CI, 32.9–51.4), followed by low back (29.9, 95% CI, 22.0–39.2) and shoulder pain (29.1, 95% CI, 21.2–38.3). Similar to helicopter pilots, a small proportion of crewmembers reported frequent pain at the neck (13.4%), shoulder (11.8%) and lower back (14.3%).

In all neck pain cases of the total group (*n* = 123), mean pain intensity during the previous 3 months was 4.7 (SD, 2.0).

Self-reported interference with flying duty was not significantly different between crewmembers and pilots (11.7 vs. 4.6%, *p* = 0.093). Although not significant, more crewmembers reported of an interference with leisure activity than helicopter pilots (27.3 vs. 24.1%, *p* = 0.646). Of all the neck pain cases (n = 123), 9.8% (95% CI, 5.4–16.9) reported that their pain negatively influenced their flying duty, while 27.0% (95% CI, 19.6–36.0) reported interference with leisure activity. Both helicopter pilots and crewmembers reported pain occurrence most common during the resting phase compared to flying (57.5 and 62.3%).

Table 1 shows the characteristics and univariate OR of potential risk factors. Neck pain cases and controls significantly differed in total flying hours (*p* = 0.001), flying hours in the previous year (*p* = 0.020) and average flying hours per month in the previous 3 months (*p* = 0.003) but not in age (*p* = 0.988), height (*p* = 0.364), weight (*p* = 0.078) and BMI (*p* = 0.242). Furthermore, more helicopter pilots suffered from flight related neck, shoulder and low back pain compared to crewmembers (*p* < 0.05).

**Table 1:** Comparison of characteristics and univariate odds ratios of potential risk factors between helicopter pilots and crewmembers suffering from flight related neck pain (cases) and those not reporting neck pain (controls)

Risk indicators	cases (*n* = 123)	controls (*n* = 98)	Odds Ratio (95% CI univariate)	*p*-value
Profession [n, %]				
Crewmember	53 (45.3)	64 (54.7)		
Pilot	70 (67.3)	34 (32.7)	2.5 (1.44–4.30)	0.001
Demographic data				
Age [years, Mean, SD]	44.7 (8.3)	44.7 (8.5)		0.988
Height [m, Mean, SD]	1.8 (0.1)	1.8 (0.1)		0.364
Weight [kg, Mean, SD]	81.5 (9.8)	80.0 (10.1)		0.078
BMI [kg/m^2^, Mean, SD]	24.9 (2.4)	24.6 (2.5)		0.242
Flying hour variables [h, Mean, SD]				
Total flying hours	3311.3 (3286.2)	2254.3 (2682.6)		0.001
Flying hours in the previous year	163.0 (134.6)	132.7 (129.0)		0.020
Average flying hours per month in the previous 3 months	25.1 (28.0)	18.3 (21.4)		0.003
NVG variables				
NVG use [n, %]				
Yes	91 (74.0)	56 (57.1)	2.1 (1.20–3.76)	0.009
NVG flying hours [h, Mean, SD]	80.3 (116.0)	46.8 (83.9)		0.071
NVG flying years [years, Mean, SD]	4.2 (3.8)	3.6 (4.6)		0.362
Helicopter type [n, %]				
Augusta Bell 58/206/212/407	9 (60.0)	6 (40.0)	1.2 (0.42–3.53)	0.726
Eurocopter 135	97 (55.7)	77 (44.3)	1.0 (0.5–1.9)	0.958
Airbus 350/355	9 (56.3)	7 (43.8)	1.0 (0.4–2.9)	0.960
Others	8 (50.0)	8 (50.0)	0.8 (0.3–2.2)	0.637
Shoulder pain [n, %]				
Yes	65 (62.8)	16 (16.3)	5.7 (3.02–10.92)	< 0.001
Low back pain [n, %]				
Yes	66 (53.7)	27 (27.6)	3.05 (1.73-5.37)	<0.001

### Multivariate results

Represented in Table 2, multivariate regression analysis revealed only one flight related factor to be significantly predictive for neck pain. The risk of suffering a neck pain episode is 1.9 fold higher (95% CI, 1.06–3.50) when using a NVG device (*p* = 0.032). Representing individual indicators, shoulder (OR 4.9, 95% CI, 2.48–9.55) and low back pain in the previous year (OR 2.3, 95% CI, 1.21–4.31) proved to be significant risk factors in the final model (p < 0.05). All other parameters (profession, demographic data, NVG variables, flying hour data and helicopter type) did not affect the onset of neck pain (*p* > 0.05).

**Table 2:** Final multivariate regression model: multivariate odds ratio of risk indicators in helicopter pilots and crewmembers self-reporting flight-related neck pain

Risk indicators	Odds Ratio multivariate	95% CI	*p*-value
Profession (pilot)	2.2	0.99–4.86	0.054
Weight (kg)	1.0	0.98–1.03	0.754
BMI (kg/m^2^)	1.0	0.97–1.05	0.548
Total flying hours (h)	1.0	1.0–1.0	0.820
Flying hours in the previous year (h)	1.0	0.99–1.00	0.586
Average flying hours per month in the previous 3 months (h)	1.0	0.99–1.02	0.291
NVG use (yes)	1.9	1.06–3.50	0.032
NVG flying hours (h)	1.0	0.99–1.01	0.102
Shoulder pain (yes)	4.9	2.48–9.55	< 0.001
Low back pain (yes)	2.3	1.21–4.31	0.011

## Discussion

Aim of the underlying study was to determine general musculoskeletal pain prevalence, particularly focusing on neck, shoulder and low back pain and to evaluate potential risk factors for neck pain among helicopter pilots and crewmembers within the Austrian armed forces, the Austrian airborne police and Austrian airborne rescue organizations.

The main finding of this study was that the 12-month neck pain prevalence is considerably high among both, helicopter pilots and crewmember (67.3 vs. 45.3%). Furthermore, the 3-month prevalence of neck pain was 64.4% for pilots and 41.9% for crewmembers, proofing the existence of this growing concern among the described occupational groups. Regardless if considering the 12- or 3-month prevalence, low back pain presents the second most common painful body part followed by the shoulder. NVG use (OR 1.9), shoulder (OR 4.9) and low back pain in the previous year (OR 2.3) seem to be the only independent risk factors for suffering from flight-related neck pain.

### NVG – Risk factor for neck pain?

Being the first study evaluating the combined 12- and 3-month pain prevalence for the whole body, our results reveal the highest musculoskeletal pain prevalence for neck pain. The results of the underlying study seem to be higher compared to results of other studies by Thomae et al. [6], Bridger et al. [7], Van den Oord et al. [38] and Van den Oord et al. [12] estimating the 12-month prevalence of neck pain among helicopter pilots and crewmembers between 29 and 62%. Murray et al. explained this difference by using more pain categories potentially resulting in higher pain prevalence [32]. While Van den Oord et al. used four pain categories (never, occasional, regular, or continuous) [38], in accordance to Murray et al. we used six categories (0 days, 1–7 days, 8–30 days, > 30 days etc.) [32].

Our evaluated 3-month prevalence of neck pain among helicopter pilots (64.4, 95% CI 54.4–73.4) was much higher compared to a study by Äng and Harms-Ringdahl, who reported of a 3-month neck pain prevalence of 57% (95% CI, 47.6–65.4) among pilots [13]. In accordance to Äng and Harms-Ringdahl, an additional time frame of 3 months was chosen to reduce potential recall bias, as more severe pain episodes may be remembered better than less serious ones [13].

Furthermore, it is to mention that in our study only 72.4% of pilots and 60.7% of crewmembers used NVG whereas in studies by Van den Oord et al. [38], Äng and Harms-Ringdahl [13], Van den Oord et al. [12] all participants wore NVG devices. Therefore, one could speculate that the underlying neck prevalence among Austrian helicopter pilots and crewmembers should have been lower. Multivariate testing showed that the use of NVG resulted in a 1.9 significantly higher risk for neck pain for the total group. No other flight related risk indicator (profession, demographic data, NVG variables, flying hour data and helicopter type) had an effect on risk. Äng and Harms-Ringdahl also found that although not significant in their final model, the use of NVG revealing users were at risk (OR 1.7) [13].

Many studies proofed, that NVG devices do not only allow pilots to enhance their visual capacity, but also come at the cost of increased mass leading to NVG-induced neck strain [21, 22]. Interestingly, in our study, helmet weight alone may have had an impact on neck pain and we refer to results of a study by Sovelius et al. who reported, that helmet weight itself seems to induce more muscle strain than NVG [39]. Higher weight of the helmet has a more significant effect on cervical muscle loading then the lighter NVG, which alters the center of gravity [39]. Moreover, neck pain prevalence might increase by using NVG more frequently, as Adam found a threshold value of 150 NVG hours, after which 90% of helicopter aircrew report neck pain [16]. In our study neck pain cases reported of 80.3 (SD, 116) NVG hours of use on average, representing a much lower limit of critical NVG use leading to neck pain.

### Flight-related neck pain – interference with flying duty and leisure activity?

The 12-month neck pain prevalence among the general adult population (17–70 years) has been estimated between 17 and 75% with a mean of 37% [11]. Therefore, our evaluated 12-month neck pain prevalence must be considered as remarkably high with potential impact on interference with leisure activity and flying duty. Interference with leisure activity was reported by 27.0% (95% CI, 19.6–36.0) of the total group whereas 9.8% (95% CI, 5.4–16.9) self-reported impairment of flying duty. These results are lower compared to results of a study by Äng and Harms-Ringdahl who reported, that 58% (95% CI, 46.1–69.9) of neck pain cases stated their pain interfered with flying duty and 55% (CI, 43.4–67.1) reported interference with leisure activity [13]. Undoubtedly, neck pain can influence operational safety [16] by limiting the level of concentration [6], motor control [26, 27] and postural stability [28]. On the other hand, a study by Aherne et al. proved, that operational safety is also influenced by other psychological factors like stress [40]. Pilots´ likelihood of night accidents resulted from pressure from the mission task, like patient condition, as one motive to continue to destination [40].

### Is flight-related neck pain the major problem?

As a novelty, we evaluated pain prevalences for the whole body and found high prevalences in other close body regions too. For helicopter pilots as well as crewmembers, regardless if considering the 12-month or 3-month prevalences, low back pain presents the second most frequent painful body region followed by the shoulder and upper back. These results correspond well with those of a study by Äng and Harms-Ringdahl [13]. It is noteworthy, that the 12-month prevalence of low back (48.1%) and shoulder pain (43.3%) within helicopter pilots were higher compared to their 3-month pain prevalence (42.3 and 38.5%).

A similar pattern was seen among crewmembers, again the 3-month prevalence of low back (29.9%) and shoulder pain (29.1%) were lower compared to the associated 12-month prevalence (36.8 and 30.8%). The questionnaire-based study was immediately conducted after the winter season (March – May), which might not constitute the peak season of work for the majority of helicopter pilots and crewmembers. This fact could have had influenced ratings of musculoskeletal disorders.

Generally, low back pain and its relation to the occupational hazards is a well-documented health problem in helicopter pilots with a life-time prevalence of 61–80% [6, 7] whereas in our study 48.1% of helicopter pilots and 36.8% of crewmembers self-reported flight-related low back pain the previous year. Furthermore, our results proved that helicopter pilots and crewmember with low back pain had a 2.3-fold higher risk to suffer from flight-related neck pain. Well in accordance with results of a study by Äng and Harms-Ringdahl [13], results showed that recent shoulder pain was a significant risk factor (OR 4.9). Many studies give evidence that previous pain and pain in other close body regions is associated with neck pain [41].

The underlying association between neck pain and pain in other close body regions gives somehow support to the argument that close body regions may share similar risk factors [41]. Obviously, flight-induced neck pain seems to be the major problem but literature on neck pain reported by helicopter pilots and crewmembers is still sparse [13]. Just recently, the issue of neck pain in helicopter aircrew has become an aeromedical concern by influencing the physical health [13, 22]. Especially in Austria, due to its topographical position, thousands of HEMS missions (approximate 6700 per year) are flown by helicopter pilots and crewmembers per year [42]. All occupational groups might have different peak seasons of work due to different operations leading to various risks suffering from musculoskeletal disorders.

Due to the increasing popularity of alpine outdoor activities in the winter as well as in the summer season, associated with a rising number of accidents [1, 43], helicopter pilots and crewmembers are under high exposure to high forces. Thus, the occupational group of rescue organization is at particular risk to suffer from flight-related musculoskeletal disorders.

### Pain frequency and intensity – is flight related neck pain a chronic ailment?

Compared to Äng and Harms-Ringdahl [13], in our study less participants reported frequent neck pain episodes (32.0 vs. 13.4%). Our sample covered helicopter pilots and crewmembers of three different occupational areas representing a wide range of job related requirements. The three occupational groups might differ within work tasks and flight manoeuvres as other studies only used air force helicopter squadrons [12, 13, 38, 44]. This could explain the higher neck pain prevalence in our study.

Generally, the majority of helicopter pilots (87.1%) and crewmembers (83.0%) in our study reported pain episodes of between one and thirty days, not supporting the previously described definition that neck pain has been described as chronic among this occupational group [44]. Similar results were found regarding shoulder and low back pain frequencies, as most of helicopter pilots (73.4% vs. 74.0%) and crewmembers (83.3% vs. 76.8%) reported of pain episodes lasting not longer than 30 days.

In all neck pain cases, the mean pain intensity during episodes was 4.7 and thus higher compared to studies by Murray et al. [32] and Äng and Harms-Ringdahl [13] who reported of mean values between 2.2 and 4.4 using a Borg Category-Ratio scale (0–10) [34]. The same scale (0 = no pain, 10 = worst possible pain imaginable pain) was used in our study. Most of the study participants belonged to the air emergency (64.0%) and had their peak season of work during summer and winter due to rescue flights in alpine terrain in Austria. This fact may have had an impact on self-reporting mean pain intensity of the previous 3 months, as the survey study was conducted between March and May.

Moreover, it is well known that military helicopter pilots and crewmembers will report a lower intensity of pain [45] compared to the general population and have been found reluctant to state pain due to fear of flying restrictions [20]. Again, these facts can influence the described prevalence and self-reported pain intensity.

### Benefits of physical exercise training for pain prevention?

The underlying results of this study highlight the need for longitudinal studies. Specific training strategies and further research is essential to reduce the pain prevalences among helicopter pilots and crewmembers. According to Sovelius et al. better muscle conditioning programs, enhanced muscle coordination and head support strategies are required to prevent neck injuries caused by the extra mass of the helmet [39].

Generally, physical exercise training has been found effective against neck pain in a number of studies conducted within other working populations [46, 47].

Only few exercise interventions, using randomized controlled study designs, have been conducted among aircrewmembers with successful outcome reducing neck pain [48, 49]. In their study, Äng et al. provide evidence that a supervised neck and shoulder exercise regimen was effective in reducing neck pain cases in helicopter pilots by improving the work capacity of muscles affected [48]. Basically, exercise training may increase individual capacity and reduce the relative workload on the cervical musculature, by further reducing the risk of developing neck pain [50]. Sovelius et al. found a benefit of trampoline training as relatively low-intensity and repetitive muscular loading improves muscle balance and motor skills [51]. Trampoline training was shown to be effective in reducing muscle strain during in-flight, especially in the cervical muscles [51]. Recapped, the hypothesis specific to helicopter pilots and crewmembers suggests training programs focus on muscular endurance and general fitness to limit the effects of cumulative exposure to multiple factors that contribute to neck pain [44].

However, there are no evidence-based guidelines and consensus regarding the prevention of flight-related neck pain among helicopter pilots and crewmembers of Austria. Thus, further randomized controlled studies are needed to clearly clarify the effectiveness of specifically tailored training interventions.

### Strengths and limitations

In the underlying questionnaire-based study, a response rate of 69.8% has been achieved. Although study participants were informed about the project at briefings, emails, by telephone and reminders were sent fortnightly, we did not achieve a higher response rate. Unfortunately, only a small number of military pilots could be acquired, presenting a potential limitation.

Pilots and crewmembers work on different air bases throughout Austria, thus to the best of our ability we tried to cover all branch establishments of the airborne police and air emergency of the whole nation. Therefore, this study can be assumed to be representative for helicopter pilots and crewmembers suffering from neck pain. We defined pain as any pain within our study potentially leading to higher pain prevalences. Furthermore as mentioned in a study by Äng and Harms-Ringdahl [13], within the limits of cross sectional studies it is difficult to estimate the direction of causality, as there is generally no evidence of a temporal relationship between exposure and outcome. Without longitudinal data, caution should be exercised in drawing conclusions about causality in any epidemiologic design, thus no true cause and effect relationship can be derived.

## Conclusions

This is the first study evaluating pain prevalences for the whole body and potential risk factors for suffering neck pain among helicopter pilots and crewmembers in Austria.

The 12- as well as the 3-month prevalence of neck, shoulder and low back pain is considerably high among both, helicopter pilots and crewmembers, proofing the existence of this growing concern among the described occupational groups. The use of NVG devices, shoulder and low back pain in the previous year present independent risk factors for neck pain. Recapped, our results concerning neck pain prevalences within helicopter pilots and crewmembers correspond well with the international trend, that flight-related and especially NVG-induced neck pain is an increasing concern among the helicopter communities. These findings highlight the need of longitudinal studies to build on these results. Trying to extend existing recommendations for pain prevention, further studies are planned to evaluate the effectiveness of specifically tailored training interventions.

## Abbreviations

BMI: Body mass index
CI: Confidence Interval
HEMS: Helicopter emergency medical services
I-CVI: Item content validity index
NVG: Night Vision Goggles
OR: Odds ratio
S-CVI: Scale content validity index
SD: Standard Deviation
